# Cerebral Small Vessel Disease in Sporadic and Familial Alzheimer Disease

**DOI:** 10.1016/j.ajpath.2021.07.004

**Published:** 2021-11

**Authors:** Rajesh N. Kalaria, Diego Sepulveda-Falla

**Affiliations:** ∗ Neurovascular Research Group, Translational and Clinical Research Institute, Newcastle University, Newcastle upon Tyne, United Kingdom; † Department of Human Anatomy, College of Health Sciences, University of Nairobi, Nairobi, Kenya; ‡ Institute of Neuropathology, University Medical Center Hamburg-Eppendorf, Hamburg, Germany

## Abstract

Alzheimer disease (AD) is the most common cause of dementia. Biological definitions of AD are limited to the cerebral burden of amyloid β plaques, neurofibrillary pathology, and neurodegeneration. However, current evidence suggests that various features of small vessel disease (SVD) are part of and covertly modify both sporadic and familial AD. Neuroimaging studies suggest that white matter hyperintensities explained by vascular mechanisms occurs frequently in the AD spectrum. Recent advances have further emphasized that frontal periventricular and posterior white matter hyperintensities are associated with cerebral amyloid angiopathy in familial AD. Although whether SVD markers precede the classically recognized biomarkers of disease is debatable, post-mortem studies show that SVD pathology incorporating small cortical and subcortical infarcts, microinfarcts, microbleeds, perivascular spacing, and white matter attenuation is commonly found in sporadic as well as in mutation carriers with confirmed familial AD. Age-related cerebral vessel pathologies such as arteriolosclerosis and cerebral amyloid angiopathy modify progression or worsen risk by shifting the threshold for cognitive impairment and AD dementia. The incorporation of SVD as a biomarker is warranted in the biological definition of AD. Therapeutic interventions directly reducing the burden of brain amyloid β have had no major impact on the disease or delaying cognitive deterioration, but lowering the risk of vascular disease seems the only rational approach to tackle both early- and late-onset AD dementia.

The most common cause of age-related dementia is the multifactorial Alzheimer disease (AD). Although <5% of biologically defined AD is thought to be familial in nature, even this proportion exhibits high phenotypic variability that can be modified by lifestyle or environmental factors. Late-onset AD is pathologically confirmed by the presence of extracellular amyloid β (Aβ) plaques, intracellular hyperphosphorylated tau, and neuron (or synaptic) loss.^1^ Early-onset or familial AD bears similar biological features, although typically, the hallmark pathology accrues considerably before 65 years of age. Clinical AD patients predominantly present with an insidious progressive irreversible amnesia and global cognitive decline in the absence of overt vascular disease. However, numerous post-mortem studies show that vast majority of patients diagnosed with AD dementia invariably have cerebral vascular pathology above and beyond normally aging healthy individuals.^2^, ^3^, ^4^ Many of the brain vascular changes are attributed to covert or silent cerebral small vessel disease (SVD). SVD features involving intracranial vessels <1 mm in diameter may be acquired subsequent to the initial clinical diagnosis of AD but most likely result from long-standing hypertensive or other vascular disease, or from age-related intracranial microvascular pathology.^5^

Though cerebral SVD independently contributes to morbidity, disability, and mortality, several studies have confirmed that SVD also adds to or modifies progression to dementia such that the threshold for impairment is reached earlier.^6^^,^^7^ For example, in the Nun study, SVD type of pathology in the form of lacunar infarcts in the basal ganglia, thalamus, or deep white matter (WM) was described to be associated with a higher prevalence of dementia.^8^ The presence of small vessel changes in clinically diagnosed and pathologically confirmed AD is not necessarily denied,^9^ but deciphering whether vascular brain injury occurs prior to or concomitantly with neurodegenerative changes, has become a burning issue in AD research.

This review discusses the current evidence of SVD in early- and late-onset AD, and evaluate its contribution in the spectrum of AD. It also appraises whether early-onset AD cases support the proposal that prior vascular dysfunction is part of the biomarker profile of AD. This is timely because it is strikingly clear that reducing brain Aβ overload via any treatment intervention has not had a substantial impact on improving cognition,^10^ but lowering the severity or risk of vascular disease seems the worthwhile option to reduce the incidence of AD dementia.

## Epidemiology and Vascular Risk Factors in AD

Although conscious efforts are made to exclude the presence of overt vascular disease in the diagnosis of AD dementia, reasonable evidence suggests that sporadic AD is associated with pre-existing or late acquired vascular risk factors. These include hypertension, diabetes mellitus, hypercholesterolemia, obesity, metabolic syndrome, and atherosclerosis; all collectively promote morbidity and specifically increase the probability of clinical AD diagnosis. Current systematic reviews and recent meta-analyses have identified hypertension in midlife, high body mass index in late life, hyperhomocysteinemia, diabetes, head trauma, and orthostatic hypotension to be most strongly associated with dementia or AD.^11^, ^12^, ^13^, ^14^ In addition, vascular risk factors promote conversion of mild cognitive impairment to frank dementia.^15^

Blood pressure is one of the most widely evaluated risk factors for dementia and AD in particular. Hypertension is a risk for WM damage as well as clinically covert lesions such as arteriolosclerosis, microbleeds, microinfarcts, infarcts, endothelial damage, and vascular inflammation.^13^^,^^16^, ^17^, ^18^, ^19^ Although nondemented, normally aging individuals have histories of hypertension, large cohort studies suggest an association between systolic hypertension (>160 mm Hg) in midlife and late-onset AD by a risk of 18% to 25%^20^ or decreased risk of AD in the short term, possibly due to reverse causation.^21^ Blood pressure variability, albeit visit-to-visit or day-to-day, is also associated with progression of AD,^22^ and optimal blood pressure management appears to be important for prevention of dementia.^23^ The case for hypertension is further supported by a recent meta-analysis that explored the association of incident AD with the use of five antihypertensive medications.^24^ High systolic blood pressure (>140 mm Hg) with a median follow-up of 7 to 22 years in those using any antihypertensive agent had reduced risk for developing AD (HR, 0.8; 95% CI, 0.7 to 1.0) compared with those not on any antihypertensive agent. There were no significant differences between one drug class versus all others on risk of dementia. This indicates that certain protective measures against cardiovascular pathology appear beneficial in AD, although the use of antihypertensives in secondary prevention of what is described as pure AD seems to be unclear.^25^ Findings from other longitudinal studies such as Atherosclerosis Risk in Communities (ARIC)^26^ and SPRINT MIND^27^ suggest that intensive blood pressure control would be beneficial for the risk of dementia and AD. Long-term hypertension may have a major role in subclinical cerebral SVD across subtypes and brain regions highlighting the need to recognize and treat hypertension early in life.^18^ It is not unlikely that long-standing increase or variability in blood pressure may disrupt vasoregulatory functions, promote blood-brain barrier (BBB) damage, and cause neurodegeneration.^28^ Each vascular factor or collectively all, in different combinations, may unevenly alter the vasculature of the body or brain to cause distortions in normal structure and function, and potentially induce a chronic cerebral hypoperfusive state in old age.^29^

Whether hypertension *per se* substantially increases AD pathology is still not clear. In the Honolulu-Asia Aging Study (HAAS), midlife systolic and diastolic blood pressures were associated with an increased number of neuritic plaques and neurofibrillary pathology,^30^ whereas clinical studies showed that higher pulse pressure was associated with cerebral amyloidosis in the presence of neurodegeneration and progression to dementia and increased plasma Aβ concentrations.^31^ Another study showed that medicated hypertensives exhibited less AD pathology.^32^ In the Religious Orders Study and the Rush Memory and Aging Project (RUSH) cohort, association between higher systolic blood pressure, albeit in late-life, was also associated with higher tangle burden.^33^ Well-designed longitudinal prospective studies with post-mortem examination and a treatment intervention to control vascular risk factors by demonstrating reduction in AD pathology and neuronal atrophy could address this question.

## Spectrum of SVD Pathology in AD

**Figure 1:**
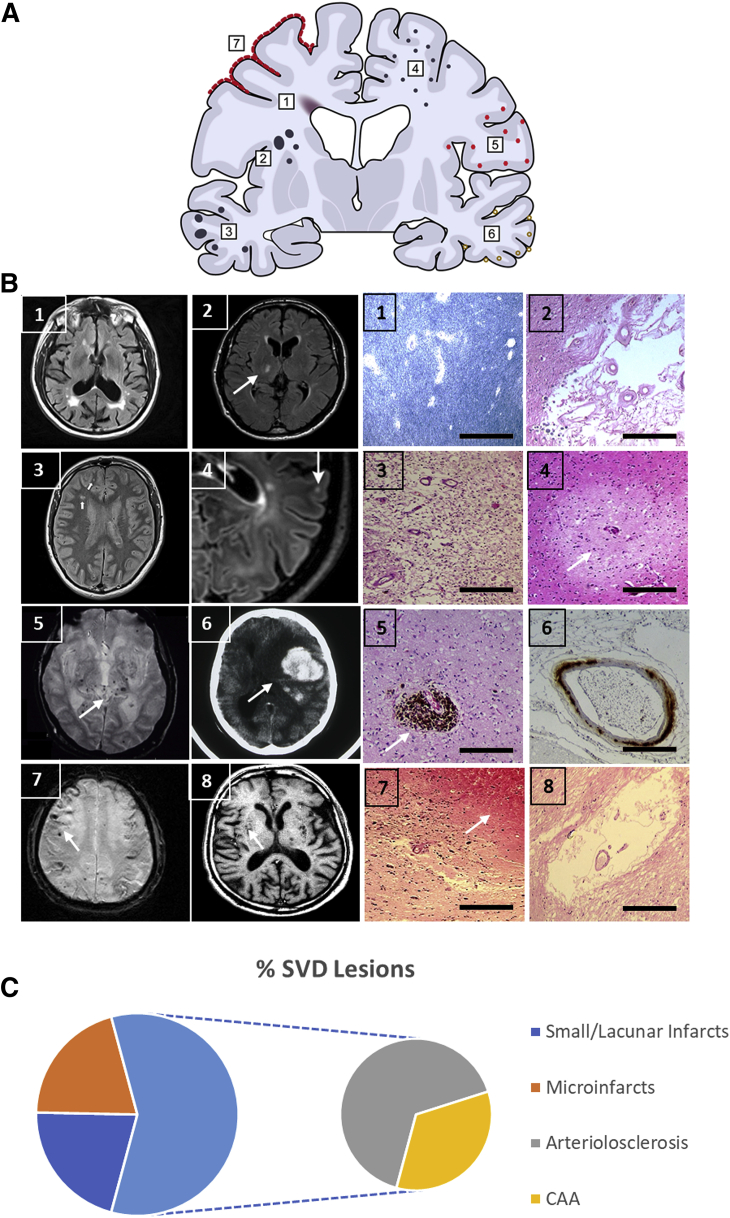
**A:** Schematic of a coronal brain section showing different types of SVD pathologies found in sporadic and familial AD. Numbers in boxes (1 to 7) correspond to the location and type of lesions in **B**. Location of perivascular spaces is generally in the WM and subcortical structures including the basal ganglia and thalamus (see image 8, axial MR scan, and corresponding histopathological image 8 on right). **B:** Images 1 to 8 (left) show MR scans in the axial plane with different lesions recognized and quantified in AD patients during life. Images 1 to 8 (right) show microscopic images of equivalent pathologies found in late-onset AD cases. Each of the pathologies has been demonstrated and often quantified in different cohorts consistently indicating greater representation of these pathologies in sporadic as well as familial AD than in aging controls or less common neurodegenerative dementias.^3^^,^^4^ These include perivascular and deep WM lesions or attenuation (image 1, right and left), lacunar infarcts (<1.5 cm) (image 2, right and left), cortical or WM infarcts (1 to 2 cm) (image 3, right and left) in a *PSEN1 Glu280Ala*, microinfarcts (<0.5 cm) (image 4, right and left), lobar or deep microbleeds or hemosiderin (image 5, right and left), CAA or CAA-related ICH (image 6, right and left), superficial siderosis (image 7, right and left), and perivascular spaces (image 8, right and left). Increased perivascular spacing occurs because of reduction in arterial vascular tone and lack of perivascular solute drainage.^35^ The WM changes ensue due to a chronic hypoxic state and decline in oligodendrocytes.^36^^,^^37^**Arrows** in panels show the location of key lesion(s). **C:** Pie chart shows average proportions of SVD pathologies in community-based observational and longitudinal cohort studies. % SVD lesions involve at least one lacunar infarct, a microinfarct, moderate-to-severe arteriolosclerosis, or CAA. Arteriolosclerosis is sometimes described as arteriosclerosis or total SVD. Perivascular spaces or WM attenuation or WM lesions were not consistently recorded. Microinfarct, unless specified, was considered that seen with the light microscope or to be <1 mm in diameter.^38^ MR fluid attenuated inversion recovery image (image 3, left) from a 57-year–old male subject was kindly provided by and used with permission from Dr. Yakeel T. Quiroz (Departments of Psychiatry and Neurology, Harvard Medical School, Boston, MA). Scale bars = 50 μm (**B**). AD, Alzheimer disease; CAA, cerebral amyloid angiopathy; ICH, intracerebral hemorrhage; MR, magnetic resonance; SVD, small vessel disease; WM, white matter.

Cerebrovascular pathology corresponding to most radiologically defined lesions is common in late-onset AD (Table 1). Although there is uncertainty whether Auguste Deter^34^ developed early- or late-onset AD, it is intriguing that Alzheimer had described vascular changes including neovascularization in this original case. Numerous studies^3^^,^^4^^,^^35^, ^36^, ^37^, ^38^ have now reported that patterns of pure neuritic plaque and neurofibrillary tangle pathology are not the norm in pathologically diagnosed AD (Figure 1). Small lesions including lacunar infarcts, microinfarcts, hemosiderin deposition or microbleeds, arteriosclerosis, cerebral amyloid angiopathy (CAA), and arteriosclerotic leukoencephalopathy, or WM attenuation, are recorded, but large infarcts (>1 cm in diameter) tend to be selected out from such cohorts.^3^ However, in a recent analysis from several centers, nearly 20% of AD subjects exhibited large infarcts.^39^ Findings from the National Alzheimer's Coordinating Centers showed that whereas only 32% of the AD cases reported cerebrovascular disease, SVD in terms of both parenchymal and vessel pathologies included lacunes in 20%, multiple microinfarcts in 20%, arteriosclerotic leukoencephalopathy in 9%, hemorrhages in 7%, atherosclerosis in 40%, arteriolosclerosis in 35%, and CAA in 41% of AD cases. Overall, various features of SVD pathology were present in up to 80% of the 4629 AD autopsies.^4^ In other clinicopathologic studies, even greater proportions of SVD pathology^40^^,^^41^ were evident. In addition, the authors have reported high frequencies (approximately 100%) of microvascular degeneration in prospectively assessed AD subjects.^42^^,^^43^ Remarkably, familial AD bears various features of SVD including severe WM attenuation and infarcts, although reporting such lesions was not the intent of previous studies, which focused on patterns of Aβ deposition in the parenchyma and intracerebral vessels.^44^^,^^45^ In fact, a large study in familial AD cases determined that WM hyperintensities (WMH) are a core feature in familial AD pathology.^46^ In a South African subject with presenilin 1 (*PSEN1*) *Ile143Met* mutation, border zone infarcts, microinfarcts, arteriolosclerosis, perivascular spaces, and severe WM attenuation were also noted.^47^ Similarly, in the large Colombian *PSEN1 Glu280Ala* kindred,^48^ severe SVD pathology not necessarily attributed to CAA was noted (D. Sepulveda-Falla, R.N. Kalaria, unpublished data).

**Table 1:** Radiological and Pathologic Features of Spectrum of Small Vessel Disease in AD Dementia

Clinical features	SVD feature	Imaging marker	Key pathologic features	Degree of change in AD (compare aging or neurological controls)∗
Stroke(s)/vascular origin	Silent infarcts	WMHs on T2W, FLAIR, ↑ high signal	Unclear	++
	Transient Ischemic attacks	WMHs on DWM; high signal	Unclear	+
	White matter attenuation	WMHs (pvWMH, dWMH), WM atrophy in CAA; high signal	Demyelination in deep WM; axon damage	+++
	Lacunar infarcts	Hyperintense lesions on T2W/FLAIR	Lacunes (<1.5 cm) in BG, thalamus, WM	++
	Cortical Infarcts	Hyperintense lesions on T2W/FLAIR	Cortical infarcts	+
	Microinfarcts	Tiny hyperintense lesions on T2W (3T, 7T)	Microinfarcts (<0.5 cm) in GM and WM	+++
	Microbleeds	T2∗W or GRE signal lobar and deep bleeds; hypointense lesions on T2W	Hemosiderin deposits in cortex (CAA) and subcortical structures (hypertensive)	++
	Intracerebral hemorrhages	Hyperintense on CT; hypointense lesions on T2W	ICH, microaneurysms	+
	Cerebral siderosis	Hypointense signal on GRE	SAH	+
Vascular pathologies	Perivascular spaces (enlarged Virchow-Robin spaces)	Hyperintense rounded lesions on T2W/FLAIR	PVS in WM; GM of BG	++
	Intracranial atherosclerosis	Unclear	Occasional microatheromas in branches of MCA, ACA	+
	Arteriolosclerosis	Unclear	Moderate-severe arteriolosclerosis	+++
	CAA	Posterior WMHs, lobar microbleeds	Moderate-severe CAA in cortex; predominance in occipital lobe	+++
Vascular Function	CBF	Resting CBF	Parietal, temporal lobes, BG	+++
	BBB function	Permeability on MRI (contrast agents, Gd)	EC damage, ↓ capillary density	+++
	PVW	Phase contrast MRI; pulse sequence with retrospective peripheral pulse gating sequences	Arteriosclerotic vessels; collagen fibers	+++
	Autonomic function (hypoperfusion)	Tilt table, carotid sinus supersensitivity (OH, CSH)	WMLs, arteriolosclerosis, microinfarcts in BG	++

Neuroimaging and pathologic changes involving SVD in AD.

ACA, anterior cerebral artery; AD, Alzheimer disease; BG, basal ganglia; CAA, cerebral amyloid angiopathy; CBF, cerebral blood flow; CSH, carotid sinus hypersensitivity; DWM, deep white matter; dWMH, deep white matter hyperintensities; EC, endothelial cell; FLAIR, fluid attenuated inversion recovery; Gd, gadolinium; GM, grey matter; GRE, gradient echo; ICH, intracerebral hemorrhage; MCA, middle cerebral artery; MRI, magnetic resonance imaging; OH, orthostatic hypotension; PVS, perivascular space; pvWMH, periventricular white matter hyperintensities; PVW, pulse wave velocity; SAH, subarachnoid hemorrhage; SVD, small vessel disease; WM, white matter; WMH, white matter hyperintensities; WML, white matter lesion.

∗ Changes found in AD type of dementia above and beyond normally aging healthy subjects. Arrow (↑) indicates increase. Scale of change means scores: +, mild (1); ++ moderate (2), severe +++ (3). Microhemorrhages may be caused by leakage by two mechanisms: microaneurysms and rupture of walls due to deposition of fibrillar proteins or iron.^28^

Besides the inherent inconsistencies in reporting of SVD lesions across different centers, there is also an apparent mismatch or perhaps under-reporting of generally low prevalence of cardiovascular disease or relevant risk factors compared with degrees of SVD pathology in AD (and other neurodegenerative disorders such as Lewy body disease).^4^ In a recent study,^49^ clinical hypertensive disease or diabetes mellitus was recorded to be 10% lower, yet pathologic features of systemic vascular disease including moderate-to-severe coronary stenosis and variable infarction were up in 50% of the cases at post-mortem examination. Subclinical disease including cardiac dysfunction increases the risk of dementia.^14^^,^^50^

Individuals diagnosed with AD in the community at large also tend to have greater vascular pathology compared with those from memory clinics.^2^^,^^51^ Normally aging community-dwelling older persons have some brain changes, and those with dementia often have multiple brain pathologies. However, cerebrovascular disease in AD patients increases the risk of developing clinical dementia and is additive,^52^ taking lower burdens of AD pathology to tip over the threshold. In the HAAS, the five most important pathologies include microinfarcts, which are clinically silent but are correlated with dementia.^53^

Recent studies have highlighted cerebral arteriolosclerosis to be common in AD and other dementias.^19^ The arteriolar wall modifications^3^ comprise fibrinoid necrosis (and/or lipohyalinosis), microatheromas, and segmental arterial disorganization (Figure 1). Focal arteriolosclerotic changes characterized by degeneration of vascular myocytes (acellular) with concentric accumulation of extracellular matrix components, such as collagen and fibroblasts, are often evident in small vessels in the deep WM and basal ganglia.^43^ Both cerebral arteriolosclerosis and atherosclerosis are independently associated with dementia and contribute to low scores in most cognitive domains, suggesting that covert vessel pathology is an under-recognized risk for AD dementia.^54^ Interestingly, the presence of apolipoprotein E (*APOE*; ApoE) ε4 allele or vascular risk factors did not change the association between either of these vascular pathologies and dementia outcome.^52^ There is also likely to be a complex, yet unappreciated, physiological interaction between risk factors associated with metabolic syndrome such as hypertension and inflammation to culminate in the arteriolar pathology.^19^

Arteriolar changes in familial AD do not appear to be vastly different from familial SVD such as those in cerebral autosomal dominant arteriopathy with subcortical infarcts and leukoencephalopathy (CADASIL).^55^ In the *PSEN1 Pro117Leu* mutation carriers, in addition to attenuation and perivascular spaces in the WM, there is an accelerated process of transformation in arteriolar myocytes characterized by loss of the tunica media, marked fibrous thickening of arteries and arterioles, and double-barreled arterioles that are comparable to, but distinct in some ways, from those in CADASIL. Cerebral capillaries with Aβ deposits also reveal enhanced expression of fibrillar collagen 3 and 4. Ultrastructural studies indicate the presence of both Aβ and collagen fibers within thickened basement membrane of capillaries. Degenerated-appearing pericytes are also observed with clusters of collagen fibers between lamellae of basement membranes.^56^ In general, these observations are consistent with other pathologic studies of familial AD.^44^^,^^47^^,^^57^

Patterns of SVD are remarkably similar in neurodegenerative diseases, and the WM is particularly vulnerable irrespective of primary ischemic injury or a proteinopathy.^3^^,^^58^ The natural history and staging of SVD suggest that arteriolosclerosis and CAA are the earliest changes. Modifications in perivascular spaces and myelin loss are the next most common lesions. Lacunar or regional infarcts, microinfarcts, and microbleeds occur because of an independent process or in the final phases of SVD (Figure 1). These may result from occlusion by microemobili or microthombi originating form artery-to-artery thromboembolism and emboli from the heart.^59^ Other causes include microaneurysms and repeated parenchymal injury resulting from the disruption of flow due to arteriolar stiffening and tortuosity. The regional progression of vessel changes suggests that CAA proceeds from neocortical to subcortical structures. Although pre-existing hypertensive disease distorts and damages the microvasculature,^19^ cerebral vessels laden with Aβ aggregates cause arteriosclerotic changes and damage the endothelium. Specific segmental patterns of capillary and arteriolar dysfunction appear to contribute to CAA and AD pathology.^42^^,^^60^ Although initiating factors causing CAA microangiopathy may be different, end-stage pathology appears invariably similar, involving replacement of myocytes with collagenous or other nontensile fibrillar material in both sporadic and familial cases. Intracranial arterial dolichoectasia also appears another cause of SVD, although this has not been widely described in AD. Microaneurysms arise in the context of hypertension, at weakened sites in vessel walls. The walls of aneurysms are composed of hyaline connective tissue, damaged myocytes, and elastica interna that may rupture to produce globular hemorrhages. They are transformed into fibrocollagenous balls, evident as complex tortuosities, when they heal due to thrombosis and fibrosis. They are most common at the interface between the grey matter and WM in most dementias.

Atherosclerosis coexists with sporadic SVD involving large extracranial vessels and cardioembolic disease. Some cohort studies have reported that atheromas within basal brain vessels are common in AD.^4^^,^^52^^,^^61^ Small vessel atherosclerosis or microatheromas are occasionally also found within proximal segments of penetrating arteries at junctions of branching and parent arteries, and in parent vessels overlying the branch origin. The pathogenesis of atherosclerosis in small cerebral vessels does not differ substantially from that in extracranial vessels but is characterized by macrophages and nearly complete stenosis.^28^ It is not surprising that subsets of proteins and modules associated with cerebral atherosclerosis were also found in AD brains.^62^

## Radiologically-Defined SVD in Late-Onset AD

WMH on T2-weighted magnetic resonance imaging (MRI) have been invariably considered as radiological surrogate markers for SVD (Figure 1). The frequency of WMH or WM lesions increases to 94% by 80 years of age. The lesions are more common and extensive in patients with cardiovascular risk factors, and increase risk of stroke, dementia, and death. Both periventricular and deep WMH of vascular origin are common in late-onset AD,^63^ and WMH and Aβ accumulation worsen cognitive outcomes.^64^^,^^65^ A recent systematic analysis indicated that extensive WMH burden was associated with higher risk of AD (HR, 1.5), suggesting that MRI markers of vascular brain injury have major clinical significance and implicate prevention strategies in individuals with covert SVD.^5^ Some frontal periventricular and posterior WMH or WM lesions are present in a large majority of familial cases of AD.^66^ In keeping with the pathology,^43^ AD subjects also have less subcortical grey matter and WM with greater volumes of whole-brain, periventricular, and deep WM subcortical hyperintensities as well as lacunar lesions.^67^

Diffusion tensor imaging has been used to assess WM microstructural integrity and assess the progression of neurodegeneration in initial stages of disease.^68^^,^^69^ Whether spatiotemporal patterns of these WM changes precede dementia symptoms in AD is uncertain. However, WM microstructural changes and increased water diffusivity in the WM in AD appear akin to SVD. Increased free water in normal-appearing WM in AD even without overt cerebrovascular disease suggests that mild vascular damage may occur due to microvascular degeneration and neuroinflammation-related BBB permeability.^70^ In addition to WMH, a repertoire of SVD features can be detected by MRI in AD that are routinely demonstrated under the microscope (Figure 1). This includes lacunar infarcts, perivascular spacing, microbleeds, and microinfarcts. Most lacunar infarcts are clinically silent, as are microinfarcts, but both are found in greater numbers in AD compared with healthy aging subjects. These lesions may gradually disrupt cognitive network, modify global cognitive performance, and cause focal atrophy. Independent of the proteinopathy in AD, ongoing studies with more sensitive 7-T MR scanners and higher resolution modalities using 3-T suggest that microinfarcts and microbleeds^71^ arise from cardiac microemboli, supporting the role of cardiovascular abnormalities in AD. Microbleeds as radiological evidence of SVD have also been detected in familial AD, specifically in patients carrying *PSEN1* missense mutations *Ala260Gly*, *Pro284Ser*, and *Pro355Ser* (AlzForum, *http://www.alzforum.org/mutations*, last accessed January 15, 2021).

Above all, the evidence for the presence of SVD in both sporadic and familial AD is compelling, although the interactions between vascular and neurodegenerative processes may not be understood. In support of the radiological findings and presence of other biomarkers of SVD, collective evidence from the Alzheimer’s Disease Neuroimaging Initiative (ADNI) studies suggests that vascular alterations such as cerebrovascular resistance and hypoperfusion precede Aβ deposition and are predictive of disease progression.^72^, ^73^, ^74^ Analysis of >7700 brain images and 1000 plasma and cerebrospinal fluid (CSF) biomarkers suggests that intrabrain vascular dysregulation is an early pathologic event during disease development. High abnormal levels of specific proteins associated with the integrity of the vascular system have been noted. This strongly implicates the inclusion of covert SVD features in the biomarker model of AD depicting disease progression. Congruent with this, brain extracellular vesicles were identified with molecular mediators of hypoxia responses and neuroprotection in preclinical AD and mixed dementias, supporting involvement of a vascular component in the etiology of AD.^75^

Elderly free of cardiovascular disease including atrial fibrillation have a lower cardiac index or output, which corresponds with lower cerebral blood flow (CBF) in the temporal lobes.^76^ In addition, cardiovascular disease including myocardial infarction in midlife is more associated with lower grey matter perfusion in older age, but not very late in life.^29^ Using advanced dynamic contrast-enhanced MRI sequences with high spatial and temporal resolutions, BBB breakdown in the hippocampus was suggested to occur in early stages of AD prior to laying down of disease pathology. This breach appears worse in individuals with mild cognitive impairment. Both clinical imaging and CSF biomarker studies indicate BBB abnormalities^77^ with reduced CBF occur 10 to 20 years prior to onset of clinical symptoms of AD.^78^ More recent studies have shown that CSF changes in markers of pericyte injury and BBB damage predict cognitive decline in patients with mild cognitive impairment independently of accumulation of Aβ or hyperphosphorylated tau.^77^^,^^79^ Thus, focal vascular dysfunction appears in early phases of AD perhaps concomitant with initial Aβ accrual. Observations from sporadic disease are consistent with earlier findings in the Colombian *PSEN1 Glu280Ala* mutation carriers; regional cerebral perfusion abnormalities detected on single-photon emission tomography occur before development of clinical symptoms.^48^ AD patients also demonstrate decreased perfusion in the posterior parietal and superior frontal cortex.^80^ In another study on familial AD, early CBF changes particularly in the lenticulostriate arterial territories were identified in asymptomatic and mildly symptomatic subjects.^81^

**Figure 2:**
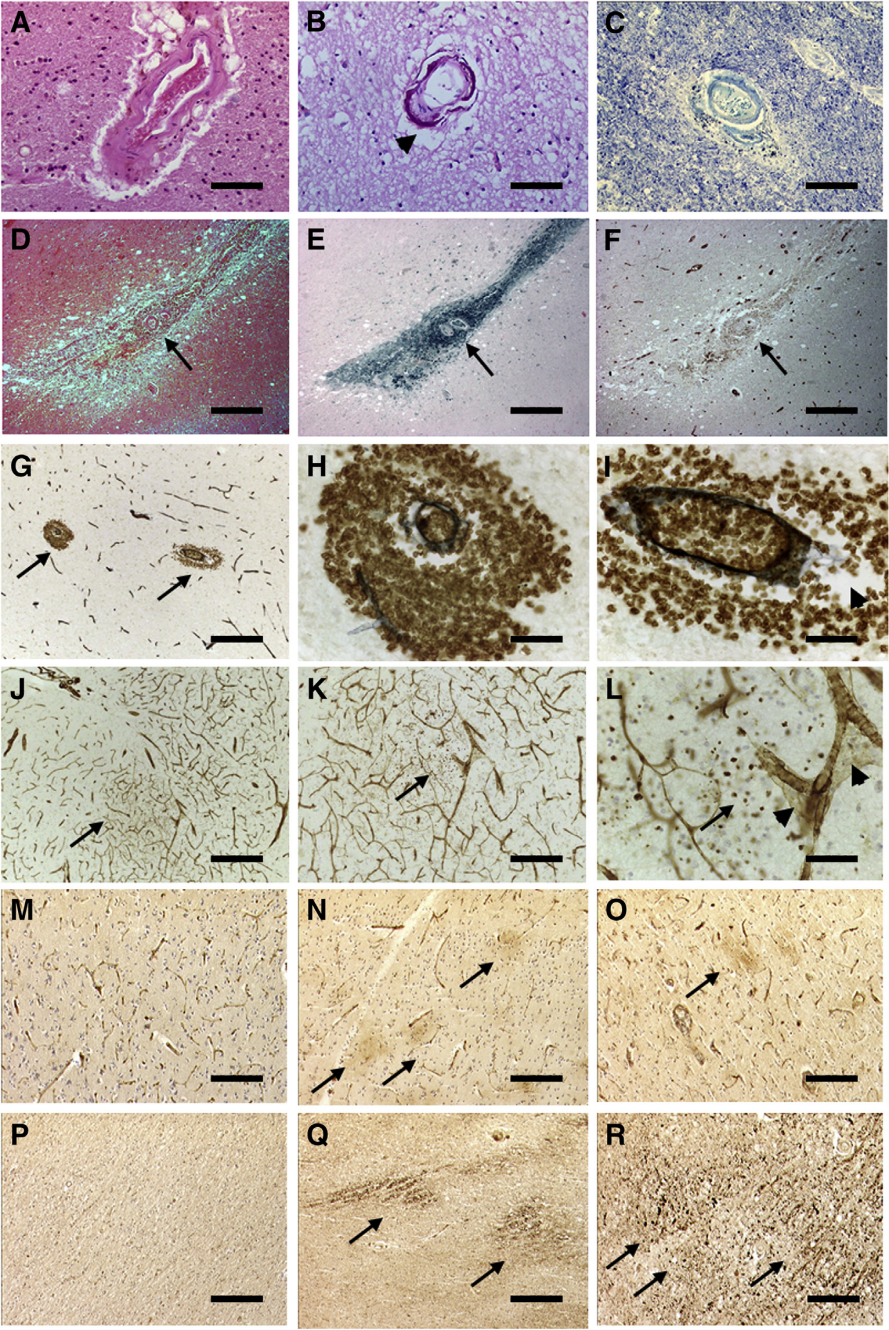
Cerebrovascular pathology in subcortical WM in AD. **A–C:** Severe arteriolar hyalinization (**A**), calcification in basal ganglia (**B**), and moderately hyalinized vessel in rarefied WM (**C**). **D–F:** Periarteriolar microhemorrhage (**arrows**) in temporal WM in a subject with CAA. Serial sections stained with hematoxylin and eosin (**D**), Perl's stain for iron (**E**), and GLUT1 antibody (brown) (**F**). **G–I**: Double immunohistochemistry (brown = GLUT1, black = COL4) shows leakage sites of GLUT1-positive erythrocytes perivascular to arterioles in the WM of an AD case. **G**: **Arrows** indicate perivascular infiltrates, shown at higher magnification in **H** and **I**. **J–L**: Double immunohistochemistry (brown = GLUT1, black = COL4) shows sites of infiltration of GLUT1-positive erythrocytes around capillaries and arterioles (**arrows**), some showing increased perivascular spaces (**arrowheads**). Comparison of images in panels **D–F** and **G–I** demonstrates differences in chronic and acute leakage from blood. **M–O**: ICAM1 IR in the WM of aging control (**M**), AD (**N**), and VaD (**O**) subjects. Increased IR in vessel walls and diffuse deposits is shown (**arrows**). Fibrinogen showed similar IR. **P–R:** APP IR in the WM of aging control (**P**), AD (**Q**), and VaD (**R**) subjects. Diffuse APP IR shows sites of damage (**arrows**). **B**, **F**, and **I**: Note the frequent perivascular spaces found in the WM in AD (**arrowheads**). Scale bars = 50 μm. AD, Alzheimer disease; APP, amyloid precursor protein; CAA, cerebral amyloid angiopathy; COL, collagen 4; GLUT1, glucose transporter 1; ICAM1, intracellular adhesion molecule 1; IR, immunoreactivity; SVD, small vessel disease; VaD, vascular dementia; WM, white matter.

The endothelium of cerebral vessels and capillaries represents a vulnerable interface, which may be chronically activated in SVD.^9^^,^^28^^,^^35^ It has been proposed that in SVD, capillary flow patterns are disrupted to retard oxygen extraction and cause SVD-like pathology and lead to neurodegeneration.^82^ However, not only hemodynamic events, but also arteriolar wall disintegration resulting from arterial stiffening, may weaken the BBB and cause chronic leakage of fluid and macromolecules (Table 2 and Figure 2). In time, this may induce an inflammatory response with increased traffic in neutrophils and lymphocytes or perivascular macrophages. In older cases, age-related decline in BBB restoration mechanisms via astrocytic response may also promote microhemorrhages in the form of perivascular hemosiderin (Figure 2). Even though so far, there is no conclusive evidence of BBB damage in familial AD independent of CAA, early or non-amyloidogenic BBB breakdown has been identified in several AD murine models involving amyloid precursor protein (*APP*), *PSEN1*, tau, and -*APOE* mutations. These findings include loss of endothelial tight junctions, basement membrane degeneration. and pericytes loss. Of particular relevance is the mice lacking *PSEN1* presenting with abnormal vessel development.^83^ This effect can be attributed to the role of γ-secretase in the cleavage and activation of Notch3, a key player in angiogenesis.

**Table 2:** Molecular Pathology of the Microcirculation and BBB in Late-Onset AD

Cellular feature	Morphological changes	Biochemical markers
Cerebral endothelium loss of glucose transporter, Na+/K+ ATPase	Loss of cytoplasm and endoplasmic reticulum. Increased pinocytosis.	↓ GLUT1, Na+/K+ ATPase, CD31, CD34
	Changes in cytoplasm (oxidative and endoplasmic reticulum stresses)	↑ Glucose-6-phosphatase; proteases (endothelin converting enzyme-1)
	Endothelial membranes/microvascular endfeet	↓ Alkaline phosphatase, γ-GGT, cholinesterases
	Decreased mitochondria	↓ Carnitine acetyltransferase
	Loss of tight junctions	
Vascular basement membrane	Thickening of the ECM, collagen fibers	↑ COL, perlecans, fibrinogen, matrix metalloproteinases
Perivascular cells	Increased astrocytic feet	↑ GFAP reactivity
	Pericytes: cell numbers and coverage	↑ CSF sPDGFRβ1; ↑ cortex PDGFRβ1cortex; ↓WM PDGFRβ1; ↑ perivascular macrophage markers, CD68, TREM2
Arteries/arterioles	Loss of vascular smooth muscle cells; increased microthrombi	↓ α-Smooth muscle actin; accumulation of Aβ
Cerebral microvessels	Changes in endothelium and perivascular macrophages	↑ Inflammatory mediators ICAM1 and cytokines

Summary of observations derived from several previous studies.^9^^,^^28^^,^^35^ Arrows indicate decreases (↓) or increases (↑).

Aβ, amyloid β protein; AD, Alzheimer disease; AlkP, alkaline phosphatase; BBB, blood-brain barrier; CD, clusters of differentiation markers; CD31, CD34 cluster of differentiation markers 31 and 34 for endothelium; CD68, cluster of differentiation marker 68 for microglia; COL, collagens; CSF, cerebrospinal fluid; EC, endothelial cell; ECM, extracellular matrix; eNOS, endothelial nitric oxide synthase; GFAP, glial fibrillary acid protein; GGT, γ-glutamyl transpeptidase; GLUT1, glucose transporter 1; HIF-1α, hypoxia inducible factor 1α; NV, neurovascular; PDGFRβ, platelet-derived growth factor receptor β; PVS, perivascular space; SMC, smooth muscle cell; TREM2, triggering receptor expressed on myeloid cells 2; VEGF, vascular endothelial growth factor; WM, white matter.

## WM Pathology and SVD in AD

Cerebral WM rarefaction or attenuation is a frequent structural change in AD (Figure 2). Periventricular WM lesions are similar to those in Binswanger's disease in up to 60% of AD.^84^ Post-mortem studies showed that patterns of loss of myelin in AD are similar to those in vascular dementia.^37^ Axonal degeneration and gliosis in the deep WM are also described to be common in AD. Similarly, other components of the gliovascular unit including capillaries undergo degeneration and dilation in the WM in AD.^58^^,^^85^ Pathologic correlates of WMH when SVD is obvious suggest demyelination, axonal abnormalities, clasmatodendrosis, microglial activation, hemosiderin deposits, arteriolosclerosis, pericyte cell loss, and BBB dysfunction secondary to degrees of vascular brain injury.^86^ In support of these morphological findings, Wong et al^87^ provided *in vivo* evidence to suggest the integrity of the BBB is compromised in relation to cerebral hypoperfusion in the WM. They reported that lower CBF was correlated with higher leakage measures in the perilesional zones, which became stronger in the proximity of WMH. However, such WM alterations cannot be fully accounted for by degenerative processes secondary to grey matter damage,^68^^,^^84^ but may stem from vascular amyloid deposition, microvascular damage, and lack of solute drainage.^88^

Diffusion tensor imaging studies in mutation carriers in familial AD indicate changes in the cerebral WM occur years before symptom onset.^89^ The mean diffusivity within the posterior parietal and medial frontal WM in mutation carriers was found to be stronger than in noncarriers. Higher mean diffusivity in fiber tracts was associated with lower grey matter volume in projection zones. These results suggest that regionally selective WM damage occurs considerably before the onset of disease that is associated with primary AD pathology and microglia activation rather than any overt vascular disease. WM changes in neurodegenerative diseases could reflect pathologic processes other than those involved in SVD, that is, that nonvascular damage could increase fluid motion in discrete areas of the WM to result in hyperintense signals.^90^ However, early changes in WM cannot all be explained by neurodegenerative pathology because there would not be sufficient burdens of neurodegenerative pathology at early stages.^1^ Is it possible then, that the presence of low perfusion or disturbed arterial pulsation within the deeper layers of the WM disrupts flow in the long perforating arteries to cause a chronic hypoxic state and damage the deep WM?

Similar to indications of BBB damage, there is still no human pathologic evidence of WM damage independent of Aβ pathology (Table 1 and Figure 2). However, alterations in myelin morphology and oligodendrocyte differentiation have been observed early in *APP*/*PSEN1* mice.^91^ Astrocytic clasmatodendrosis has shown to be a pathologic correlate of WM damage.^85^ Accordingly, the authors have observed this feature in the WM of *PSEN1 Glu280Ala* cases (D. Sepulveda-Falla, R.N. Kalaria, unpublished data).

## Consequences of Chronic Vascular Disease

Besides age, hypertension and diabetes mellitus are among the strong risk factors for SVD. Whereas it is not fully understood how diabetes might lead to SVD,^92^ the deleterious effects of increased blood pressure are mediated by structural changes in smaller arteries leading to arteriolosclerosis with two main consequences. First, the progressive segmental loss of myocytes with replacement by collagen fibers reduces vessel wall tone or elasticity in response to variations in blood pressure and loss of autoregulation through disruption of the perivascular nerve plexii. Second, the persistent high pulse pressure leads to focal disruption of capillaries, particularly in the deeper structures. This causes edema and BBB breach with chronic leakage of fluid and macromolecules (Figure 2) as well as incidental infarction, particularly in subcortical structures.

Aging-associated central arterial stiffness may increase SVD features with consequences on progression in the AD continuum. For example, aortic stiffening conveyed by higher pulse wave velocity, and therefore higher pulsatility, was associated with lower CBF particularly in the temporal lobes although cerebrovascular reactivity was preserved in *APOE* ε4 allele carriers with mild cognitive impairment.^93^ Consistent with this, higher systolic blood pressure and pulse pressure,^94^ or diastolic blood pressure^95^ attributable to arterial stiffness, were related to greater cerebral retention of Pittsburgh Compound-B (PiB) in presymptomatic and AD patients. Arterial stiffness, or the surrogate marker pulse wave velocity, and mean arterial pressure were also highest in individuals with both high PiB retention and WMH (double hit), promoting the development of AD.^96^^,^^97^ Although resting CBF may not be affected, higher pulsatility index likely promotes larger WMH volumes and increases perivascular spaces in subcortical structures.^98^ It is conceivable that early in the presymptomatic stage prior to the manifestation of disease phenotype, vascular reactivity is compromised due to covert changes in vessel walls whether they are CAA or AD mutation carriers.^99^

Rodent models of Aβ amyloidosis including those exhibiting CAA have demonstrated impairment in different features of cerebrovascular function including CBF, functional hyperemia, and cerebral autoregulation.^100^ Although higher cerebrovascular resistance and altered transfer of CBF to cortical oxygenation in AD suggests that the microcirculation and properties of the microvasculature are changed,^101^ all functional measures are not consistently replicated in AD. For example, it is controversial whether autoregulation in AD is altered *per se* to reflect in SVD-related cerebral perfusion. In an earlier study, Zazulia et al^102^ reported there was absence of significant change in CBF with a 10 to 15 mm Hg reduction in mean arterial pressure within the normal autoregulatory range, suggesting that there was no generalized or local defect of autoregulation. However, using a linear mixed model, a recent study^103^ demonstrated that the efficacy of cerebral autoregulation, assessed during stepwise changes in arterial pressure, was reduced in individuals with amnestic mild cognitive impairment, which is a prodromal stage of AD. Various limitations including cohort sizes, coexisting factors, imaging techniques, variability in disease progression and study power can be attributed to inconsistent findings.

## SVD and CAA in Sporadic and Familial AD

For more than 100 years, cerebral congophilic angiopathy, or now widely described as CAA, has been identified as a pathologic hallmark of brain disease.^104^ CAA was originally identified to be associated with wall thickening of small and medium-sized vessels together with the occurrence of cerebral microbleeds. Vessel wall thickening characteristically showed accumulation of eosinophilic material, Congo Red positive, identifying it as amyloid. The most common type of CAA is Aβ angiopathy, which is reported to be as high as 90% in AD.^105^ The majority of pathologically verified familial AD subjects exhibit CAA.^47^^,^^56^^,^^106^, ^107^, ^108^, ^109^, ^110^, ^111^, ^112^, ^113^, ^114^, ^115^, ^116^, ^117^, ^118^, ^119^, ^120^, ^121^, ^122^, ^123^, ^124^, ^125^, ^126^, ^127^, ^128^, ^129^, ^130^, ^131^, ^132^, ^133^, ^134^, ^135^, ^136^, ^137^, ^138^, ^139^, ^140^, ^141^, ^142^, ^143^, ^144^, ^145^, ^146^, ^147^, ^148^, ^149^, ^150^, ^151^, ^152^, ^153^ The first case in Argentina with marked vascular pathology including CAA carried the *APP Ala171Thr* mutation.^154^ Specifically, at least 16 mutations in the *APP* and *PSEN1* genes are outright known for CAA in the clinical phenotype (Table 3). However, of 68 mutations identified in *APP*, 15 of them present with CAA. *APP* mutations are localized between amino acids 670 to 694 and 713 to 717. The first cluster corresponds to α- and β-secretase cleavage sites and the second cluster to γ-secretase cleavage sites, indicating a direct role of abnormal Aβ peptide(s) generation and their accumulation in cerebral vessels. Several murine AD models developed from these *APP* mutations have indicated CAA as well and have favored research in AD vascular pathology. So far, more than 320 *PSEN1* mutations have been identified. Accordingly, 37 *PSEN1* missense mutations are associated with the presence of mild-to-severe CAA (Table 3). These mutations are evenly distributed alongside the *PSEN1* gene sequence. However, *PSEN1* mutations below codon 200 have been characterized as having milder CAA pathology, whereas *PSEN1* mutations above codon 200 characteristically show severe CAA. Finally, while 64 mutations for *PSEN2* have been identified, at least 4 mutations have been associated with the presence of CAA (Table 3). It has been suggested that the composition of Aβ aggregates differs between parenchymal deposits and those found in CAA-affected vessels. Parenchymal deposits are typically composed of longer Aβ peptides, often with additional posttranslational modifications. CAA deposits, on the other hand, show shorter forms of Aβ peptides.^155^ Mutations in *APP*, *PSEN1*, and *PSEN2* genes modify size and biochemical profiles of Aβ peptides generated from *APP*. The authors have shown that familial AD cases also show a distinctive Aβ peptide signature in CAA deposits when compared with those from sporadic AD cases.^142^

**Table 3:** Familial AD Causative Mutations for CAA

Gene	Mutation∗	CAA†	Notes‡	References§
*APP*	*LysMet670/671AsnLeu*	Yes	Mouse model, CAA at 12–19 months	106
	*Ala673Val*	Yes		107
	*Asp678His*	Yes	Also present with cerebral microvasculopathy (23931937)	108
	*Ala692Gly*	Yes		109
	*Glu693Gly*	Yes	Mouse model shows no CAA	106
	*Glu693Lys*	Yes		110
	*Glu693Gln*	Yes	Mouse model, CAA at 12–22 months	111
	*Asp694Asn*	Yes	Mouse model: *APP*SwDI (Swedish *Lys760Asn/Met671Leu, Dutch Glu693Gln* and Iowa *Asp694Asn*), considered to be the optimal CAA model	112
	*Ala713Thr*	Yes	WM changes, cerebral microangiopathy and CAA‖	113,154
	*Thr714Ile*	Yes	Transgenic *APP*695 mouse harboring *Lys670Asn, Met671Leu,* and *Thr714Ile*, develops CAA	114
	*Ile716Phe*	Yes		106
	*Val717Phe*	Yes	Mouse model shows no CAA	115
	*Val717Gly*	Yes		116
	*Val717Ile*	Yes	Mouse model, CAA at 15 months	117
	*Val717Leu*	Yes		118
*PSEN1*	*Ile83_MetM84del*	Yes	(*DelIleMet, ΔIle83/Met84, ΔIle83/ΔMet84*)	119
	*Met84Thr*	Yes		120
	*Val89Leu (G>T)*	Yes		121
	*Leu113_Ile114insThr*	Yes	(*Intron4, p.113+1delGly, splice5, InsThrAlaCys*)	122
	*Leu113Glns*	Yes		123
	*Thr116Asn*	Yes		124
	*Pro117Leu*	Yes		56
	*Glu120Gly*	Yes		125
	*Asn135Tyr*	Yes		126
	*Met139Val*	Yes		127
	*Ile143Met*	Yes	CAA prominent in meningeal vessels	47
	*Ile143Val*	Yes		128
	*Leu174Met*	Yes		129
	*Glu184Asp*	Yes		130
	*Ile202Phe*	Yes		131
	*Gly217Asp*	Yes		132
	*Leu219Pro*	Yes		133
	*Aal260Gly*¶	?		134
	*Val261Phe*	Yes		135
	*Gly266Ser*	Yes		136
	*Pro267Ala*	Yes		137
	*Leu268Pro*	Yes		138
	*Arg269His*	Yes		139
	*Leu271Val*	Yes		140
	*Val272Ala*	Yes		141
	*Arg278Ile*	Yes		127
	*Glu280Ala*	Yes	Paisa mutation	142
	*GluE280Gly*	Yes		143
	*Leu282Val*	Yes		144
	*Pro284Leu*	Yes		145
	*Leu286Pro*	Yes		138
	*Ser290Cys*	Yes	*Thr291_Ser319del* (ΔAla9, Δ9)	146
	*Gly378Glu*	Yes		123
	*Leu392Val*	Yes		145
	*Asn405Ser*	Yes		147
	*Gly417Ser*	Yes		148
	*Ala431Val*	Yes		145
	*Thr440del*	Yes		149
*PSEN2*	*Aal85Val*	Yes		150
	*Lys115Glufs*	Yes		151
	*Asn141Ile*	Yes	Volga German mutation	152
	*Leu221Thr*	Yes		153

AD, Alzheimer disease; *APP*, amyloid precursor protein; CAA, cerebral amyloid angiopathy; *PSEN1*, presenilin 1; *PSEN2*, presenilin 2; WM, white matter.

∗ Genotypes of different mutations per original references derived from the Alzforum Database (*http://www.alzforum.org/mutations*, last accessed January 15, 2021).

† Presence of variable degrees of CAA predominantly in cortical regions.

‡ Presence or absence of CAA in various transgenic mouse models and features noted in case reports.

§ References include citations of four abstracts.

¶ Magnetic resonance imaging positive for microbleeds suggests likely CAA (compare Figure 1B, images 5 and 6).

‖ First case in Argentina with *APP Ala171Thr* mutation showing marked vascular pathology.^154^

In sporadic AD, CAA is more common in individuals with infarction and hemorrhages, and is also recognized as an independent factor for severe cognitive impairment and dementia. SVD pathology in CAA is characterized by progressive segmental arteriolosclerosis involving the medial-adventitial layers of intracranial arteries. Of the two types of CAA, Type I is associated with capillaries implicating focal BBB damage. Pathologic studies have shown variable patterns of CAA between early-onset and late-onset AD; Type I CAA is more common and more severe in *APP* duplication and missense mutations and in Down's syndrome compared with those in sporadic early- and late-onset AD.^44^ That CAA plays a role in the pathogenesis of microvascular lesions is important,^40^^,^^156^ but it is not the only factor, implying that even in familial AD, microvessels may undergo age-related changes prior to the appearance of CAA and independent of Aβ accumulation.^88^ For example, cerebral hypoperfusion associated with WM damage accelerates CAA and promotes cortical microinfarcts.^157^

In early-onset autosomal-dominantly inherited AD patients, ischemic WM changes can be detected at least 2 decades before the development of dementia. The Dominantly Inherited Alzheimer Network (DIAN) study indicated that mutation carriers had greater total WMH volumes, which appeared to increase approximately 6 years prior to expected symptom onset.^46^ Given the propensity for a posterior distribution of WMH, CAA, which tends to be more prominent in the occipital lobe, was thought to be one of the mediating factors. These findings suggest that WMH are an essential feature of AD and should be incorporated into the biomarker model and a potential therapeutic target.^46^^,^^158^

Besides posterior dominant WMH, the radiological features of CAA include lobar microbleeds, dilated perivascular spaces, and multiple areas of superficial siderosis (Figure 1). Mutation carriers are more likely to have cerebral microbleeds than noncarriers, and patients with microbleeds have higher WMH volumes. Although there is some codependency between WMH and microbleeds, these observations highlight that WMH represents a core feature of AD independent of vascular forms of Aβ.^158^ In another imaging study, the peak skeletonized mean diffusivity, a measure of cerebral WM microstructural disruption as a simple marker of diffuse global WM heterogeneity, was increased in CAA suggesting a role for WM disruption in causing cognitive impairment in CAA.^159^

## Conclusions and Future Directions

**Figure 3:**
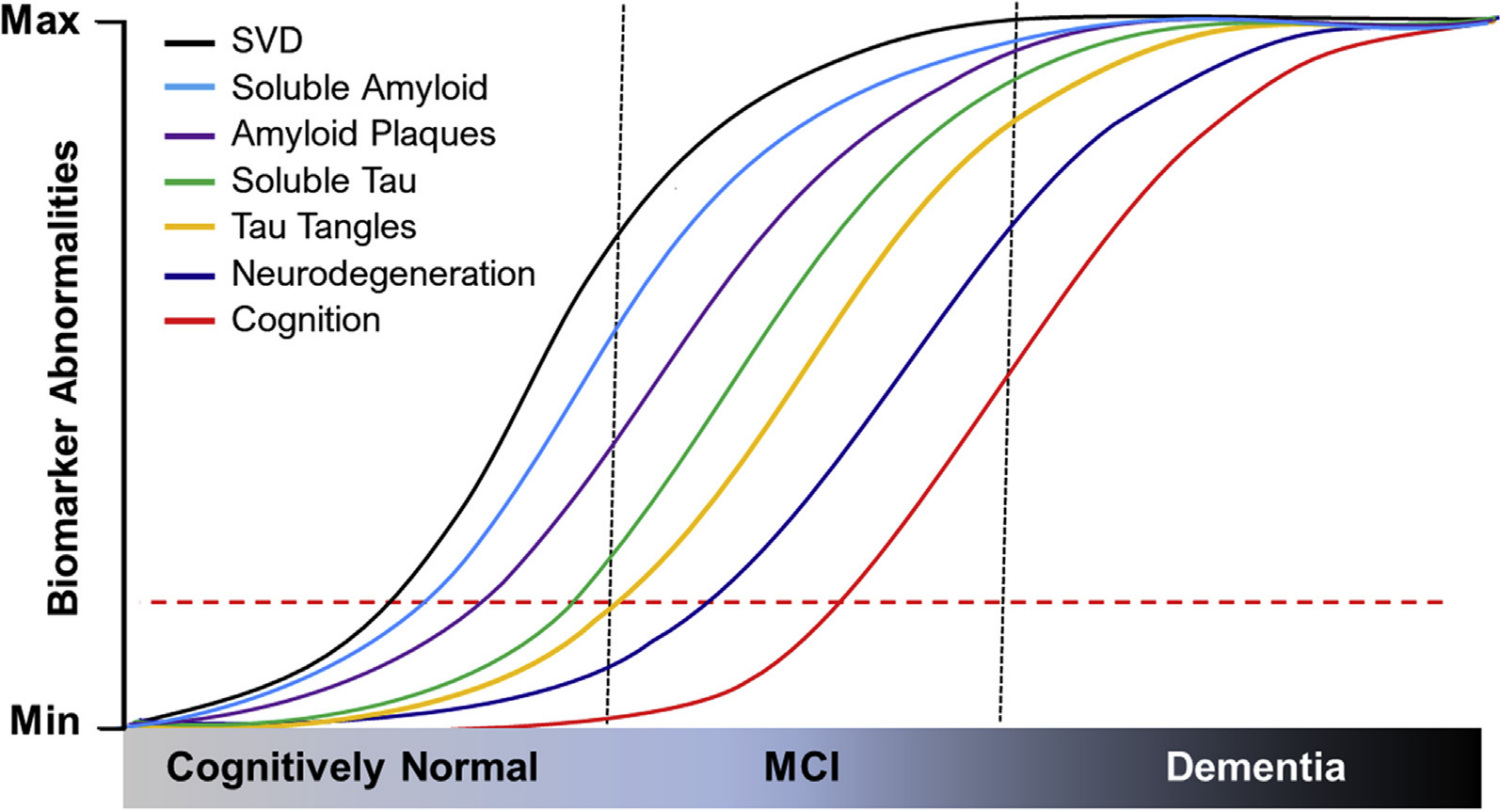
Proposed modification of risk factors and biomarkers of AD in older age. Early changes include vascular disease factors associated with SVD in the progression of AD. SVD incorporates covert vascular brain injury including arteriolosclerosis in the early phase that may alter cerebral perfusion to cause WM changes and lead to the recognized biomarkers of AD. Fluctuations in CBF, blood pressure variability, perfusion pressure, PWV, and BBB damage may contribute to prodromal stages in the AD continuum. Vascular factors increase conversion of mild cognitive impairment (MCI) to dementia in the form of frank or clinical AD. These changes also promote changes in CSF and brain Aβ and tau pathophysiology. The *y* axis represents increasing severity or accumulation of the biomarkers. The **horizontal dashed line** represents lower threshold changes in markers with minimal impact on clinical symptoms. Neurodegeneration is depicted as a late phase, but it is likely that tissue atrophy occurs concomitantly or as a consequence of SVD or small infarct pathology.^28^ Aβ, amyloid β protein; AD, Alzheimer disease; BBB, blood-brain barrier; CBF, cerebral blood flow; CSF, cerebrospinal fluid; Max, maximum; Min, minimum; PVW, pulse wave velocity; SVD, small vessel disease; WM, white matter.

There is a wealth of evidence from neuroimaging and pathologic studies demonstrating that various features of cerebral SVD are inherent to the AD continuum. The presence of greater burdens of SVD pathology in normal aging are evident, not only in late-onset AD, but also in mutation carriers with familial AD. Thus, vascular brain injury and consequent tissue changes in both grey matter and WM, particularly in the latter, are the norm rather than the exception. It is further apparent that vascular lesions or SVD modify the progression of disease and increase the odds of dementia (Figure 3). Hypertension-induced arteriosclerotic disease is an important index in AD as is CAA, which is irrefutably conditional to age-related changes in the cerebral intracranial vasculature. It is thought that vascular brain injury occurs concomitantly with neurodegenerative changes and that these are parallel processes without much mechanistic interaction between them. The fallacy of this argument is that in many studies, vascular brain injury used as a surrogate for SVD is assessed by overt lesions, such as infarcts,^28^ evident both radiologically and pathologically. However, it is more likely that clinically silent lesions or covert changes characteristic of SVD contribute to precipitate AD type of pathology over long periods of time (Figure 3). Robust proof of this and of vascular risk factors including history of hypertension increasing the burden of Aβ or neurofibrillary pathology are generally lacking. It is plausible that some of the evidence is confounded by selection bias of participants whose vascular risk factors are well-controlled, and markers of SVD such as WMH may not be evident in those at risk.^160^ However, such evidence can be derived from large, comprehensive, prospectively followed longitudinal studies where SVD features including indices assessing BBB damage^77^ are carefully monitored in both individuals at risk and those in the early stages of clinical disease. Evidence of early SVD and WM damage in familial AD reinforces the certainty of AD as a multifactorial disorder. From this perspective, it is conceivable that the presence of SVD is either part of the pathologic processes that lead to AD, or that in some patients, SVD coexists with some other possible AD trigger, lowering the threshold for cognitive dysfunction and eventual dementia. It is probable that other cellular effects of familial AD mutations independent of Aβ deposition also contribute to early SVD in familial AD cases (Figure 3). In the absence of any strong support for therapeutic approaches that lower Aβ deposition or reduce neurofibrillary pathology, it is timely to strongly implement therapeutic and preventative measures that improve or retain functional properties of cerebral small vessels in the context of AD pathologies including CAA.
